# Optimized Morphology and Tuning the Mn^3+^ Content of LiNi_0.5_Mn_1.5_O_4_ Cathode Material for Li-Ion Batteries

**DOI:** 10.3390/ma16083116

**Published:** 2023-04-15

**Authors:** Yan Lin, Juho Välikangas, Rafal Sliz, Palanivel Molaiyan, Tao Hu, Ulla Lassi

**Affiliations:** 1 Research Unit of Sustainable Chemistry, Faculty of Technology, University of Oulu, 90570 Oulu, Finland; 2 Kokkola University Consortium Chydenius, University of Jyvaskyla, 67100 Kokkola, Finland; 3 Optoelectronics and Measurement Techniques Unit, University of Oulu, 90570 Oulu, Finland

**Keywords:** LiNi_0.5_Mn_1.5_O_4_, sol-gel method, Mn^3+^ content, cathode materials, li-ion battery

## Abstract

The advantages of cobalt-free, high specific capacity, high operating voltage, low cost, and environmental friendliness of spinel LiNi_0.5_Mn_1.5_O_4_ (LNMO) material make it one of the most promising cathode materials for next-generation lithium-ion batteries. The disproportionation reaction of Mn^3+^ leads to Jahn–Teller distortion, which is the key issue in reducing the crystal structure stability and limiting the electrochemical stability of the material. In this work, single-crystal LNMO was synthesized successfully by the sol-gel method. The morphology and the Mn^3+^ content of the as-prepared LNMO were tuned by altering the synthesis temperature. The results demonstrated that the LNMO_110 material exhibited the most uniform particle distribution as well as the presence of the lowest concentration of Mn^3+^, which was beneficial to ion diffusion and electronic conductivity. As a result, this LNMO cathode material had an optimized electrochemical rate performance of 105.6 mAh g^−1^ at 1 C and cycling stability of 116.8 mAh g^−1^ at 0.1 C after 100 cycles.

## 1. Introduction

The development of the battery manufacturing ecosystem is crucial to the ambitious worldwide push toward renewable energy resources and electric vehicles (EVs). High energy density lithium-ion batteries (LIBs) are urgently needed to meet the soaring demand for diverse portable gadgets, hybrid electronic devices (HEVs), and electric vehicles (EVs) [1,2,3,4]. Since cathode materials are responsible for a significant portion of the weight and expense in state-of-the-art LIBs, the development of low-cost and high-performance cathode is a considerable research direction for next-generation LIBs [5,6,7,8,9]. Currently, commercial cathodes, such as those made of lithium cobalt oxide (LiCoO_2_) and its derivatives LiNi_x_Mn_y_Co_1−x−y_O_2_ (NMC) and LiNi_x_Co_y_Al_1−x−y_O_2_ (NCA), are commonly utilized, although the cost of metallic cobalt materials (70,000 USD per tonne) has increased dramatically [10,11,12,13]. Therefore, many kinds of cobalt-free materials, including LiFePO_4_ (LFP), LiNiO_2_ (LNO), LiMn_2_O_4_ (LMO), and LiNi_0.5_Mn_1.5_O_4_ (LNMO), have also been investigated to produce cathode materials that are abundant and affordable [14,15,16,17]. As is known to all, the energy density of LIBs depends not only on the specific capacity but also on the operating voltage. To achieve high energy density LIBs, it is effective to create high-voltage cathode materials. Spinel LNMO stands out for its high operating voltage platform (4.7 V vs. Li/Li^+^) and high energy density (~650 Wh kg^−1^). However, the disordered LNMO suffers from the intrinsic defect of the presence of Mn^3+^ and oxygen vacancies due to the high-temperature calcination. Due to the disproportionation reactions (2Mn^3+^ → Mn^2+^ + Mn^4+^), the presence of Mn^3+^ leads to transition metal dissolution and Jahn-Teller distortion, resulting in structural instability and capacity decay [18,19,20].

The most popular synthesis techniques for LNMO are the sol-gel, co-precipitation, and solid-state reaction approaches [21,22,23]. Among them, the sol-gel method is frequently employed due to its advantages of product uniformity, low cost, and easy operation. During the sol-gel synthesis, the inorganic raw components are thoroughly combined, and a stable, transparent sol is created after a series of hydrolysis and condensation chemical processes [24]. The sol is slowly polymerized between the colloidal particles to form a gel with a 3D network structure. Furthermore, the 3D channel lattice structure allows quick diffusion routes for lithium ions in three dimensions [25]. Finally, the gel is dried, sintered, and solidified to prepare the final micron- or even nanometer-sized powder material. The particle size and microscopic morphology of the materials significantly impact the electrochemical performance. In various studies, in-depth research has been conducted on the impacts of pre-calcination temperature, calcination temperature, and heating/cooling rate on electrochemical performance. Okudur et al. investigated the influence of pre-calcination temperature on the morphology and electrochemical performance of LNMO particles [26]. Liang et al. studied the impact of changing calcination temperature on particle size [27]. However, the effect of reaction temperature during the sol-gel method has mainly gone unnoticed. Various reaction temperatures have been used in reports on the synthesis of LNMO compounds. Lin et al. investigated the effects of Mg and Y doping on the electrochemical performance of LNMO using 140 °C as the reaction temperature [28]. With 70 °C serving as the reaction temperature, Nisar et al. reported the impact of SiO_2_ coating on the electrochemical performance of LNMO [29]. The results demonstrate that alterations in reaction temperature led to different LNMO particle morphologies, significantly affecting electrochemical performance.

Inspired by the above literature, herein, we synthesized high-voltage spinel LNMO by a citric acid-assisted sol-gel method. We investigated the effects of reaction temperature on the physical properties of LNMO materials and the electrochemical performance of coin cells (half cells) for LIBs using LNMO as the cathode. The synthesized materials were characterized by X-ray diffraction (XRD), Scanning Electron Microscope (SEM), X-ray photoelectron spectroscopy (XPS) analysis, and electrochemical performance evaluations. The results revealed that the LNMO material obtained under the condition of 110 °C performed the most homogeneous morphology as well as a lower Mn^3+^ content, which further improved the electrochemical performance of the material.

## 2. Materials and Methods

### 2.1. Materials and Synthesis

Spinel LNMO materials were synthesized using a citric acid aided sol-gel method. First, solution A was obtained by 18.91 g of citric acid as a chelating agent dissolved in 20 mL of ethylene glycol (EG, VWR, Helcinki, Finland) with magnetic stirring in an oil bath at 90 °C for 1 h. Next, stoichiometric lithium acetate (LiAc·2H_2_O, ACROS, 98%), manganese acetate (MnAc_2_·4H_2_O, ACROS, 99+%), and nickel acetate (NiAc_2_·4H_2_O, ACROS, 99%) were sequentially added to 100 mL of deionized water (DI) with magnetic stirring at room temperature until the chemicals dissolved completely to obtain solution B, where the amount of total metal ion is equal to that of citric acid. It should be pointed out that an excess of 5% lithium acetate was added to compensate for the loss of lithium during the elevated calcination step. Subsequently, solution B was added to solution A and then heated at *x* °C (*x* = 80/110/140/170) with continuous stirring until getting a viscous gel. The viscous gel was dried overnight at 105 °C to obtain a dry gel. The dried gel was preheated at 500 °C for 6 h to remove organic components. Finally, the LNMO material was obtained after further annealing at 900 °C for 12 h in an air atmosphere followed by grinding, labeled as LNMO_*x* (*x* = 80/110/140/170).

**Figure 1:**
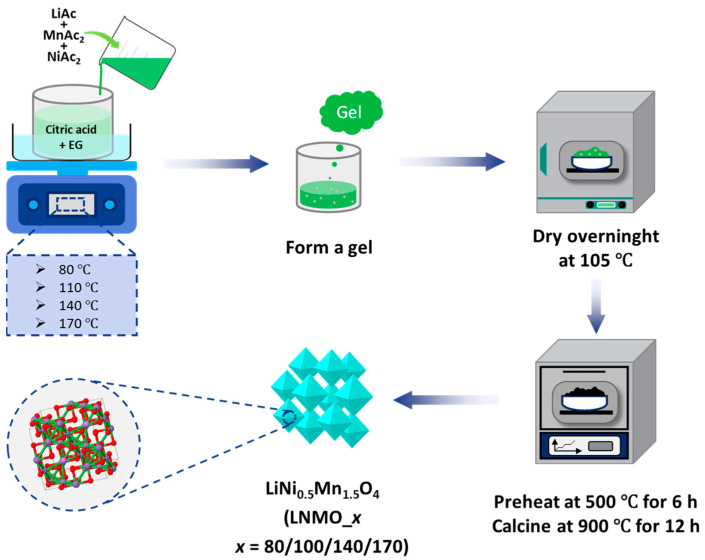
Illustration of the synthesis process of LNMO_*x* samples.

Figure 1 shows the schematic illustration of the LNMO materials preparation process. In this synthetic procedure, temperature control played a crucial role in crystal formation. The oil bath temperature was adjusted to manage the material’s morphology, particle size, and Mn^3+^ content. Gels were created at various temperatures and varying lengths of time in the oil bath. Finally, LNMO crystals were prepared after drying and calcination.

### 2.2. Characterizations

The surface morphology was detected by a Zeiss Sigma Field Emission Scanning Electron Microscope (FESEM, Carl Zeiss Microscopy GmbH, Jena, Germany) operated at 5 kV. The particle size aggregation phenomenon was further measured by a laser diffraction particle size distribution (PSD) analyzer (Malvern Mastersizer 3000, Malvern, UK). The concentration of the elements was measured by inductively coupled plasma optical emission spectrometry (ICP-OES) using an Agilent 5110 VDV ICP-OES (Santa Clara, CA, USA) equipped with an SPS 4 autosampler. 

The crystal phases and structures were identified using Rigaku SmartLab 9 kW X-ray diffraction (XRD, Rikagu Corporation, Tokyo, Japan). The XRD diffractograms were measured using a cobalt slit as a source at 40 kV and 135 mA and collected in the 2θ range of 5–120° at a step of 0.01° intervals and scan speed of 4.06 deg/min. Rietveld refinement was used to analyze the obtained powder diffraction data by the Rigaku PDXL2 (version 2.8.4.0) analysis software package. The peaks were identified using International Centre for Diffraction Data ICDD (PDF-4+ 2022). 

Chemical environment at the surface area was conducted by X-ray photoelectron spectroscopy (XPS) analysis using a Thermo Fisher Scientific ESCALAB 250Xi XPS System (Thermo Fisher Scientific, 168 Third Avenue, Waltham, MA 02451, USA). The powder samples were placed on a gold sample holder. The high-resolution scan used pass energy of 20 eV, while the Survey scan used pass energy of 150 eV. The monochromatic Al Kα radiation (1486.7 eV) operated at 20 mA and 15 kV with an X-ray spot size of 900 µm. The Li, Ni, Mn, O, and C were measured for all samples, and the measurement data were analyzed by Avantage V5 program. The charge compensation was carried out by applying the C1s at 284.8 eV as a reference to determine the presented spectra and calibrate the binding energies. The FESEM, XRD, and XPS analysis were performed at the Centre for Material Analysis, University of Oulu (Oulu, Finland).

### 2.3. Electrochemical Tests

Electrochemical performance tests were performed using a 2016-type coin half-cell. For the working electrode, 92% active material (LNMO), 4% polyvinylidene fluoride (PVDF, Kureha #1100, Tokyo, Japan), and 4% carbon (Super C, Timcal C45, Bodio, Switzerland) with 1-methyl-2-pyrrolidinone (NMP, Alfa Aesar, anhydrous 99.5%, Haverhill, MA, USA) as solvents were prepared by mixing using a mixer (Thinky ARE-250, Tokyo, Japan). The slurry was evenly spread on the aluminum foil with a thickness controlled at 100 μm. After drying on a 50 °C hot plate for 1 h, the foil was put into a vacuum oven to dry overnight at 120 °C. Before assembling the coin cells, the cathode foil was calendered three times and then cut into discs with a diameter of 14 mm. The active material loading of each disc was about 8.4–9.1 mg cm^−2^. A metallic lithium foil (Alfa Aesar, 99.9%, 0.75 mm foil) was used as the counter electrode, and 1M LiPF_6_ in 1:1:1 EC:DEC:DMC (Novolyte Technologies, Suzhou, China) served as the electrolyte. All the cell assemblies were performed in the dry room with a room temperature of 25 °C. The theoretical capacity of LNMO used to calculate the C-rate was 147 mAh g^−1^, and the voltage range of the tests was 3.5–4.9 V. The Electrochemical Impedance Spectroscopy (EIS) measurements were conducted using the Arbin LBT21084UC system connected with the Gamry Instruments 1010E Potentiostat/Galvanostat. The measurements of fresh coin-cell batteries were performed at a frequency range of 1 MHz–10 mHz, and an amplitude of 10 mV. The conductivity measurements were performed by measuring the conductivity of the LNMO samples placed between two steel electrodes in a non-conductive tube (8 mm diameter) and pressed with a pressure of 100 kg/cm^2^. Consequently, HIOKI IM3590 Chemical Impedance Analyzer was used to acquire the resistance of the samples. The measurement was repeated ten times for each sample, and the results were averaged.

## 3. Results and Discussion

**Figure 2:**
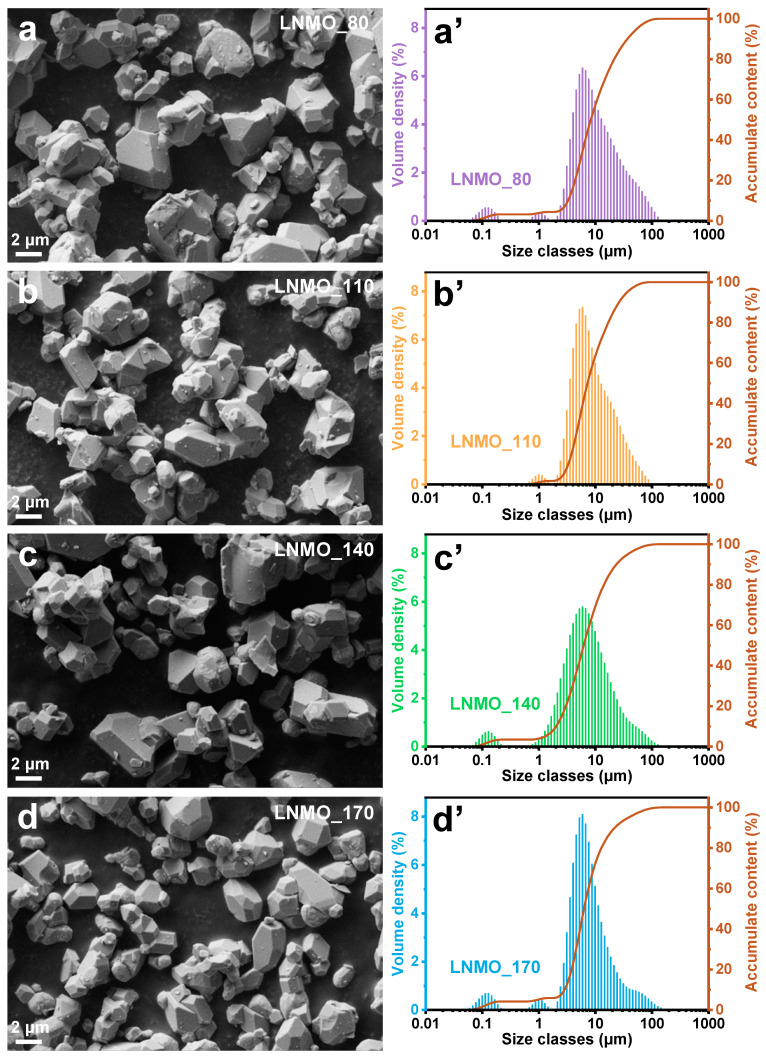
(**a**–**d**) SEM images and (**a’**–**d’**) PSD diagrams of LNMO_*x* samples.

Figure 2 shows the SEM images (Figure 2a–d) and corresponding PSD diagram (Figure 2a’–d’) of LNMO materials obtained from different synthesis temperatures. All samples have a rock-like morphology with a micrometric size that can be seen in SEM images (Figure 2a–d). Obviously, the LNMO particles display a regular crystal shape with a clear edge and smooth surface, whose size mainly ranges from 1 μm to 20 μm. Additionally, more large (>20 μm) and small (<1 μm) particles are observed in the LNMO_80, LNMO_140, and LNMO_170 materials. In contrast, the LNMO_110 sample demonstrates a homogeneous particle distribution. It is also indicated by the PSD characterization (Figure 2a’–d’). The first peak around 0.1 μm of the LNMO_80, LNMO 140, and LNMO_170 materials are observed in Figure 2a’,c’,d’, representing the small particles in the materials. The narrow peak and high peak value and accumulated content curve corresponding to the range distribution of the LNMO_110 material can be seen in Figure 2b’. Moreover, the D_99_ value of the LNMO_110 material is 68.1 μm, and that of the LNMO_80, LNMO_140 and LNMO_170 materials are 99.5, 82.5 and 91.2 μm, respectively (Table S1), which demonstrates the relatively narrow and uniform size distribution of LNMO_110 material. These results are consistent with the SEM observations in Figure 2a–d. Based on our extensive previous research, we have found that different particle distributions can result in distinct packing densities and that optimal particle distribution can lead to higher tap densities and an increased number of active sites, ultimately resulting in improved electrochemical performance [10,11,30].

**Figure 3:**
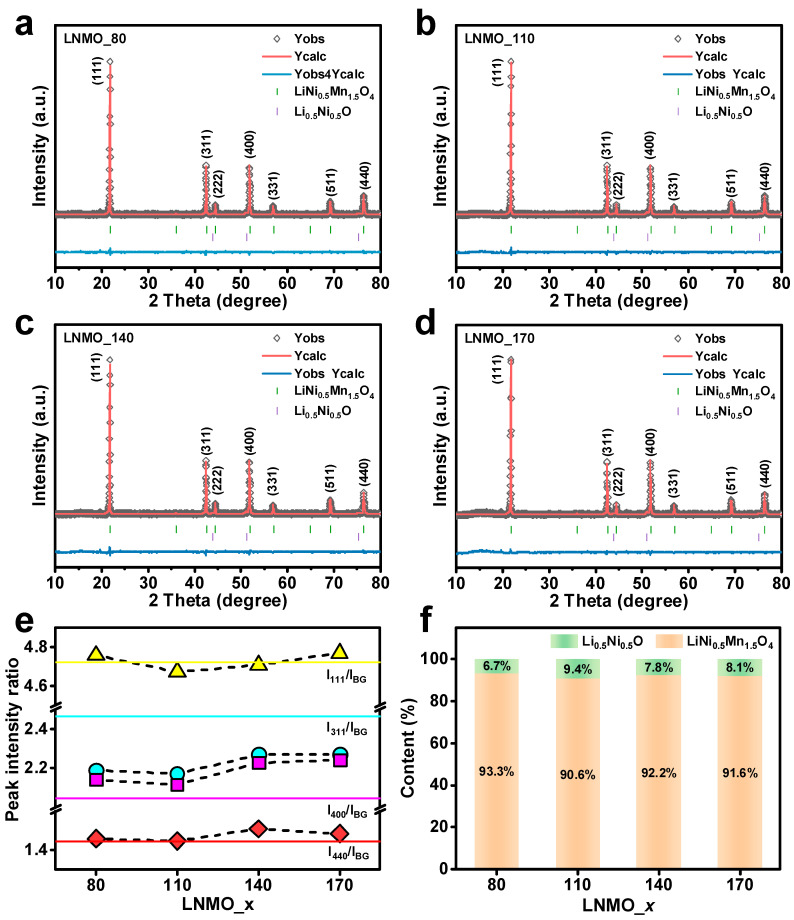
(**a**–**d**) XRD patterns and Rietveld refinement comparison of LNMO_*x* samples. Bragg positions of the LiNi_0.5_Mn_1.5_O_4_ phase and Li_0.2_Ni_0.8_O phase are shown with vertical bars. (**e**) Peak intensity ratios of (111), (311), (400), and (440) peaks to the mean intensity of the background. The corresponding horizontal line is the expected value from the PDF standard card of pure LNMO. (**f**) Quantitative analysis from profile-fitted peaks by RIR method.

Figure 3a–d display the XRD patterns and Rietveld refinement profiles of the synthesized LNMO_*x* samples. The XRD patterns revealed distinct well-matched peaks of the cubic spinel LiNi_0.5_Mn_1.5_O_4_ phase (LNMO, ICDD/PDF4#01-080-2984) with the Fd-3m space group in addition to a trace amount of an impurity of the rock-salt Li_0.5_Ni_0.5_O phase (LNO, ICDD/PDF4#04-007-6739), which is typically present in LNMO [27]. The spinel and rock salt phases, which are commonly denoted as AB_2_O_4_ and AB_2_O_3_, can be reversibly transformed by the following equations regarding the oxygen loss during synthesis [31].

(1) $${\mathrm{AB}}_{2}O_{4}\overset{0.5O_{2}}{\Leftrightarrow }{\mathrm{AB}}_{2}O_{3}$$

**Figure 4:**
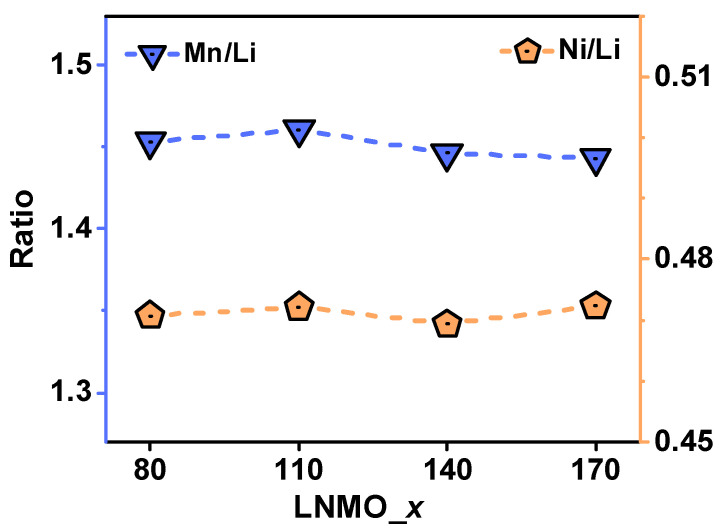
The atomic ratio of the Mn and Ni to Li within the LNMO_*x* samples based on ICP-OES measurement results.

Furthermore, studies have also demonstrated that the existence of this phase was due to the reduction in a part of the Mn in the LNMO from Mn^4+^ to Mn^3+^ during high-temperature calcination to maintain the charge balance, while the disordered phase is formed due to the loss of Li [32]. Rietveld refinement was performed on the XRD diffractograms to calculate lattice parameters (Table S2). Compared with LNMO_80 and LNMO_170, LNMO_110 and LNMO_140 have smaller lattice parameters; this phenomenon may be due to the larger radius of Mn^3+^ (0.6500 Å) than that of small Mn^4+^ (0.5300 Å) [33]. Certainly, the influence of nickel ions should also be considered, and further elemental analysis will be discussed in the XPS section. It means oxygen loss was more severe, and a higher Mn^3+^ concentration appeared in the sample. The (111), (311), (400), and (440) peak intensities were quantified as intensity ratios relative to the mean intensity of background (I_BG_) of the LNMO_*x* samples, as shown in Figure 4e. The I_111_/I_BG_ peak intensity ratio of LNMO_110 is the lowest, indicating the sample has relatively low crystallinity. Furthermore, all ratios fluctuated within a small range, possibly due to changes in LNMO particle morphology and size [26]. The components were quantitatively analyzed using the Reference Intensity Ratio (RIR) method, as shown in Figure 3f. The ratio of LNO in LNMO_110 was higher than in the other three samples, which proved that more oxygen was lost during calcination and more oxygen vacancies were present in the sample, which is beneficial to electron transfer and electrochemical performance [34]. This was confirmed by ICP-OES results, as shown in Figure 4 and Table S3. The lowest Li concentration and the highest Mn/Li and Ni/Li ratio of LNMO_110 revealed more Li volatilization for this sample.

**Figure 5:**
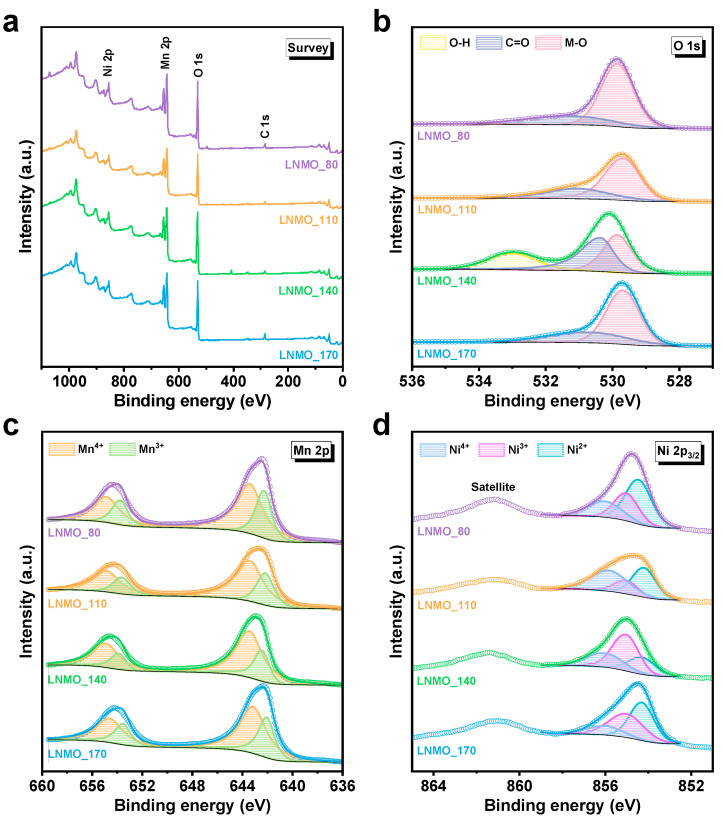
XPS spectra of LNMO_110 sample: (**a**) full survey, (**b**–**d**) high-resolution spectra of O 1s, Mn 2p and Ni 2p_3/2_.

XPS was performed to further investigate and understand the surface chemical state of the LNMO_*x* samples. Figure 5a displays the full elemental survey, which showed that all samples were composed of C, O, Mn, and Ni elements, where C should be derived from the test environment. O 1s spectra showed that all four samples included two peaks located at 529.8 eV and 531.2 eV were attributed to lattice oxygen (M–O) and surface oxygen (C=O), respectively, dominated by lattice oxygen (Figure 5b) [35,36]. Additionally, LNMO_140 existed at a peak of 533.0 eV which was ascribed to the O–H bond from adsorbed contamination [7]. The binding energy of Mn 2p showed two obvious peaks at 654.6 eV and 642.7 eV, corresponding to Mn 2p_1/2_ and Mn 2p_3/2_, which can be deconvoluted into four peaks (Figure 5c). Among them, two dominant peaks centered at 654.9 eV and 643.4 eV belonged to Mn^4+^, which was from pure LNMO [6]. Additionally, two peaks at 653.8 eV and 642.3 eV corresponded to Mn^3+^. As no satellite feature of MnO was shown between Mn 2p_1/2_ and Mn 2p_3/2_, the presence of Mn^2+^ was not considered. A proper concentration of Mn^3+^ is beneficial to the electrochemical performance of the materials [37]. Figure 5d shows Ni 2p_3/2_ spectra, which is convoluted into three peaks centered at 856.3 eV, 855.6 eV, and 854.7 eV, corresponding to Ni^4+^, Ni^3+^, and Ni^2+,^ respectively (Figure 5d) [38]. The presence of trace amounts of Ni^4+^ and Ni^3+^ was supposed to be the partial oxidation from Ni^2+^ and the existence of LNO [33]. Different concentrations of Mn and Ni elements and valence states were calculated based on XPS results, as shown in Table 1. LNMO_140 showed the least Mn^3+^ content, which is beneficial to mitigate the disproportionation reaction of Mn^3+^ in the electrochemical cycle and improve the structural stability, thus improving its electrochemical performance [39]. 

**Figure 6:**
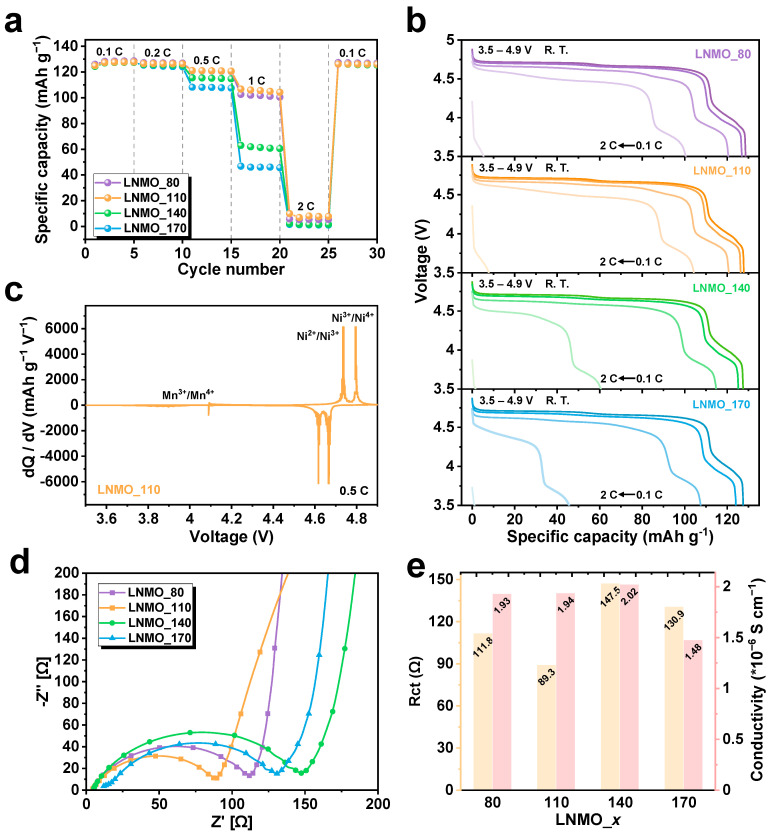
(**a**) Rate performance and (**b**) Discharge profiles at different rates of LNMO_*x* samples. (**c**) Differential capacity curves of LNMO_110. (**d**) EIS curves of fresh cells and (**e**) R_ct_ fitting values and conductivity results for LNMO_*x* samples.

The influence of different particle sizes and morphology on electrochemical performance was explored in the Li-ion half cells. Figure 6a shows the rate performance of LNMO_*x* samples at various C-rates from 0.1 C to 2 C and then return 0.1 C. LNMO_110 exhibited the best rate performance, especially at relatively high current density. Four samples all had similar specific capacities at low current density, such as ~128.5 mAh g^−1^ at 0.1 C and ~126.5 mAh g^−1^ at 0.2 C, respectively. Four samples showed distinct performance differences when the current density was increased to 1 C. LNMO_110 exhibits the optimal performance of 105.6 mAh g^−1^ at 1 C, higher than that of LNMO_80 (102.0 mAh g^−1^), LNMO_140 (61.4 mAh g^−1^) and LNMO_170 (46.2 mAh g^−1^). This is mainly due to the more uniform particle distribution of LNMO_110 and the presence of a moderate amount of Mn^3+^ and oxygen vacancies favouring ion transport, resulting in better performance [40]. When the current density returned to 0.1 C, the capacity still delivered a capacity of ~126.6 mAh g^−1^, indicating that the materials had good reversibility. Long-cycle performance was also detected at the current density of 0.2 C and 1 C, as shown in Figure S1. LNMO_110 showed a high specific capacity of 116.8 mAh g^−1^ at 0.1 C after 100 cycles and 40.0 mAh g^−1^ at 1 C after 90 cycles, higher than those of the other three samples. It should be pointed out that there is a 0.5 C rate of current density every 20 cycles after stabilization to check the battery activity so that the capacity increases every 20 cycles in Figure S2b. Galvanostatic discharge profiles at different rates of LNMO_*x* samples were compared in Figure 6b. Two characteristic plateaus around 4.7 V were inspected for all four samples, corresponding to the Ni^2+^/Ni^3+^ and Ni^3+^/Ni^4+^ redox reactions [41]. Simultaneously, a small plateau at 4.0 V came from the Mn^3+^/Mn^4+^ redox couple [42]. Figure 6c shows the differential capacity curves, which can reflect small changes that are not easily found on the voltage curves. Two anodic peaks at around 4.75 V area and two cathodic peaks at around 4.65 V area were observed, originating from Ni^2+^/Ni^3+^, and Ni^3+^/Ni^4+^ redox couples, respectively. The broad peak around 4.0 V originated from the Mn^3+^/Mn^4+^ redox pair, revealing the presence of the Ni/Mn disordered phase, which was beneficial to improve the kinetics of the material and thus contributed to exhibiting better electrochemical performance [31]. A comparative analysis of the prepared LNMO material with other previously reported pure LNMO electrodes was performed, as presented in Table 2. The electrochemical kinetics were further investigated through Electrochemical Impedance Spectroscopy (EIS) and conductivity measurements. The Nyquist plots obtained from EIS measurements and the R_ct_ fitting values and conductivity results were presented in Figure 6d,e. Among the tested samples, LNMO_110 exhibited the lowest R_ct_ value of 89.3 Ω and a relatively high powder conductivity of 1.94 × 10^−6^ S cm^−1^, indicating low resistance on the interface and high conductivity. Therefore, LNMO_110 demonstrated the best electrochemical performance. 

## 4. Conclusions

In summary, high voltage spinel LNMO were successfully synthesized by the citric acid-assisted sol-gel method as the cathode materials of LIBs. The particle size distributions and morphologies of obtained LNMO materials were adjusted by different reaction temperatures. SEM and PSD analysis confirmed that the micrometer-sized LNMO obtained through the condition of 110 °C possessed the most homogeneous distribution and morphology. The XPS analysis of the element environment on the surface revealed the presence of Mn^3+^, with the LNMO_110 sample exhibiting the lowest Mn^3+^ content of 28%. This lower Mn^3+^ content is expected to benefit Li^+^ diffusion during the charge-discharge process, resulting in enhanced electrochemical properties. Indeed, the LNMO_110 sample demonstrated improved cycling and rate performance, achieving 116.8 mAh g^−1^ at 0.1 C after 100 cycles and 105.6 mAh g^−1^ at 1 C. Further studies focus on the stability of high-voltage electrolytes with LNMO compatibility and long cycle optimizations for LIBs.

## Figures and Tables

**Table 1:** XPS and calculated results of Mn and Ni.

Sample	Mn			Ni			
	Mn^3+^/%	Mn^4+^/%	Valence	Ni^2+^/%	Ni^3+^/%	Ni^4+^/%	Valence
LNMO_80	32.2	67.8	3.678	48.9	28.1	23.0	2.741
LNMO_110	27.7	72.3	3.723	23.8	50.1	26.1	3.023
LNMO_140	29.1	70.9	3.709	39.2	17.6	43.2	3.040
LNMO_170	34.1	65.9	3.659	44.8	39.7	15.4	2.706

**Table 2:** Electrochemical performance comparison with previously reported pure LNMO materials.

Sample Name	Current Density (C)	Specific Capacity (mAh g^−1^)	Cycle no.	Ref.
LiNi_0.5_Mn_1.5_O_4_	0.3	130.6	300	[43]
LNMO-900	0.1	135	50	[44]
LNM-0.25	1	119	200	[45]
LiNi_0.5_Mn_1.5_O_4_- 5.0 °C/min	1	102	100	[46]
1000 °C-40 h	0.2	134	50	[47]
D-700	0.1	101.4	50	[31]
LNMO_110	0.1	116.8	100	This work

## Data Availability

No additional data is covered in this article.

## Supplementary Materials

The following supporting information can be downloaded at: https://www.mdpi.com/article/10.3390/ma16083116/s1, Table S1: Particle size distribution of LNMO_*x* samples; Table S2: Structure parameters of LNMO_*x* materials; Table S3: Metal analysis of LNMO samples measured by ICP-OES.; Figure S1: Cycling performance of LNMO_*x* materials at the current densities of 0.2 C (**a**) and 1 C (**b**).
