# Supplementary material for: Optimized Morphology and Tuning the Mn3+ Content of LiNi0.5Mn1.5O4 Cathode Material for Li-Ion Batteries
Source: Materials (Basel). 2023 Apr 15;16(8):3116. doi: 10.3390/ma16083116 (PMC10142292; doi:10.3390/ma16083116)
Supplement: Supplementary file 1 [file materials-16-03116-s001.zip › materials-2319821-supplementary.pdf]

Supporting Information

Optimized Morphology and Tuning the Mn^{3+} Content of $LiNi_{0.5}Mn_{1.5}O_4$ Cathode Material for Li-Ion Batteries

Yan Lin [1,*], Juho Välikangas [1,2], Rafal Sliz [3], Palanivel Molaiyan [1], Tao Hu [1] and Ulla Lassi [1,2,*]

[1] Research Unit of Sustainable Chemistry, Faculty of Technology, University of Oulu, 90570 Oulu, Finland
[2] Kokkola University Consortium Chydenius, University of Jyvaskyla, 67100 Kokkola, Finland
[3] Optoelectronics and Measurement Techniques Unit, University of Oulu, 90570 Oulu, Finland
* Correspondence: yan.lin@oulu.fi (Y.L.); ulla.lassi@oulu.fi (U.L.)

Table S1. Particle size distribution of LNMO_*x* samples

	D_x (1)	D_x (10)	D_x (50)	D_x (90)	D_x (99)
LNMO_80	0.109	3.72	9.42	41.8	99.5
LNMO_110	1.12	3.76	8.25	29.6	68.1
LNMO_140	0.114	2.29	7.05	26.5	82.5
LNMO_170	0.1	3.24	7.15	25	91.2

Table S2. Structure parameters of LNMO_*x* materials.

Sample	*a*-axis [Å]	*c*-axis [Å]	Volume [$Å^3$]	R_p	R_{wp}	R_{exp}	*S*	χ^2
LNMO_80	8.17634 (13)	8.17634 (13)	546.609	0.59%	0.78%	0.67%	1.1576	1.3400
LNMO_110	8.17555 (8)	8.17555 (8)	546.451	0.63%	0.82%	0.66%	1.2403	1.5384
LNMO_140	8.17513 (7)	8.17513 (7)	546.366	0.66%	0.88%	0.67%	1.3173	1.7354
LNMO_170	8.17619 (10)	8.17619 (10)	546.579	0.79%	1.15%	0.67%	1.7204	2.9597

Table S3. Metal analysis of LNMO samples measured by ICP-OES.

Sample name	Li ($mg \cdot g^{-1}$)	Mn ($mg \cdot g^{-1}$)	Ni ($mg \cdot g^{-1}$)	Li (mol-%)	Mn (mol-%)	Ni (mol-%)
LNMO_80	38.1	438	152.0	34.2	49.7	16.1
LNMO_110	38.0	439	151.5	34.1	49.8	16.1
LNMO_140	38.2	438	151.6	34.3	49.6	16.1
LNMO_170	38.6	441	154.0	34.3	49.5	16.2

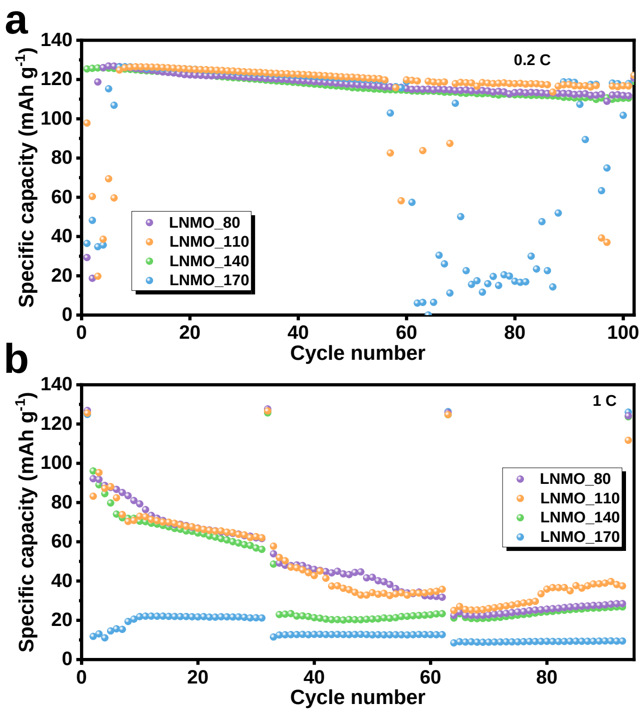

Figure S1. Cycling performance of LNMO_*x* materials at the current densities of 0.2 C (a) and 1 C (b).
